# Migrant physicians’ unlocking of gateways to practise their knowledge: A qualitative quasi-longitudinal study

**DOI:** 10.1371/journal.pone.0282317

**Published:** 2023-03-15

**Authors:** Linda Sturesson Stabel, Magnus Öhlander, Terese Stenfors

**Affiliations:** 1 Department of Learning, Informatics, Management, and Ethics, Karolinska Institutet, Stockholm, Sweden; 2 Department of Ethnology, History of Religions and Gender Studies, Stockholm University, Stockholm, Sweden; Shandong University of Science and Technology, CHINA

## Abstract

This study explores the establishment experiences of physicians in the Swedish medical context who have been trained outside the European Union. The study used a qualitative approach with a quasi-longitudinal research design. The data were gathered via 63 semi-structured interviews with migrant physicians at three different periods. The data were analysed using qualitative thematic content analysis, adopting the theory on the context dependence of knowledge, which includes different forms of knowledge as sensitising concepts in the discussion. The MPs perceived themselves as having the medical knowledge (encoded knowledge) needed to work in Sweden. However, they perceived that they needed to develop knowledge of how to use the encoded knowledge in the Swedish medical context. The needed knowledge was thus foremost encultured, embedded, embodied, or embrained. The results are presented in the following themes: *medical knowledge; knowledge of the healthcare system and its variations; knowledge of administrative routines; understanding the role as a physician*, *interaction and hierarchies between physicians and other healthcare staff; understanding the interaction and hierarchies between physicians and patients; and knowledge of the Swedish language*. Knowledge, as described in the themes, function as gateways that needs to be unlocked for practising medicine in a new context. Embedded, embrained, embodied, and encultured knowledge interact and are interdependent, and the different forms of knowledge work as gateways to other forms of knowledge, and thus, they open for each other. However, to pass the gateways, managing the common language is important. We conclude that language is an enabler and a key to unlocking gateways to practise.

## Introduction

Physicians who have trained abroad and migrated often need to undergo a recertification process before they start practising in the destination country. For example, physicians trained outside European Union/European Economic Area (EU/EEA) must take part in such a process to practise in Sweden. Before their new establishment, migrant physicians (MPs) can be interpreted as having a peripheral position (cf. [1–3]), and to become part of the new medical context, MPs may need to (re)contextualise, transfer, and translate their knowledge (4). This move of highly skilled workers and their knowledge from one cultural and organisational context to another is a multidimensional process [4]. In addition to transferring existing knowledge, MPs may also need to develop new knowledge; however, the line between transfer and development is very fine [3]. Being established in the labour market is important not only for the individual but also for society. If MPs knowledge is utilized, they can fill staff shortages [5, 6].

Knowledge transfer processes may run smoothly or may be difficult [4]. Research has shown difficulties for MPs during their recertification processes, establishment, and advancement within a destination country’s medical labour market. Difficulties may relate to a time-consuming recertification process and many years since practising, but foremost to differences in context. Some difficulties are considered cultural or have cultural characteristics [5, 7–10], and thus relate to human interaction and concern the relationships between MPs and patients, peers, other healthcare professionals, and gender [8, 9, 11–15], including how to approach and work together [5, 7–10, 15]. Another difference that often leads to difficulties during the recertification process and for workplace integration is language [5, 7, 16–20]. Language barriers may affect communication between the MP and the patient, peers, and other health professionals [7, 9]. Differences related to country-specific healthcare systems and organisations may also be challenging, including, for example, laws and guidelines, as well as other treatments and names of drugs [6, 15, 16, 21]. To bridge any differences and support MPs’ transitions, educational interventions are sometimes developed; these may include cultural and country-specific information, language and communication training, laws, guidelines, and medical knowledge [5, 11, 16, 18, 22–25]. However, research based on MPs’ own perceptions of what ‘they need to learn, and how they are supported, are often distant from those of the educators responsible for planning their education’ [26].

The knowledge needed may vary depending on the MPs’ position in the destination country’s medical context: at the beginning of the recertification process or later. Research about MPs usually captures only one snapshot, and scholars have therefore advocated longitudinal studies about the professional integration of highly trained migrants, such as physicians [27]. Research with a longitudinal perspective on how MPs’ knowledge is aligned to a destination country’s medical context, and any knowledge needs and lasting difficulties are thus scarce, as is information about whether the lack of some knowledge stalls possibilities to transform or develop other knowledge. Missing in research is thus a perspective that exposes long-lasting needs and difficulties.

In this article, we use theory of the context dependence of knowledge, which includes different forms of knowledge [2, 3, 29]. We understand knowledge as created in an ongoing process and thus being under constant development, contextualised, and socially situated [2, 3, 28]. According to Blackler and Williams, knowledge may take different forms: encultured, embedded, embodied, embrained, or encoded [2, 3, 28, 29]. Some forms of knowledge (encultured and embedded) may be place-specific and only partly transferable to another context. Other forms of knowledge (embrained and embodied) are easier to transfer between settings, as these forms are encapsulated in the individual [3]. Migration is one channel for transferring knowledge between contexts and involves a combination of embrained, embodied, encultural, and embedded knowledge [3]. These forms interact and are related to tacit knowledge, while the form of encoded knowledge is explicit knowledge. Encoded knowledge is embedded in, for example, textbooks, signs, and symbols [3, 30]. With this as a theoretical framework, MPs’ experiences of establishment in the Swedish medical context are explored using a quasi-longitudinal approach. The aim of this study is to identify the knowledge that MPs trained outside the EU/EEA perceive they need to develop over time to work as physicians in Sweden.

## Materials and methods

The study used a qualitative approach with a quasi-longitudinal research design. Data were gathered via semi-structured interviews with physicians who had a medical degree from outside the EU/EEA. The interviews were conducted at different stages of their establishment in the Swedish medical context.

### Context: The route to the Swedish medical licence and the complementary programme

MPs in Sweden have three different routes to obtaining the Swedish medical licence. To begin any route, MPs must have formal qualifications in the Swedish language. One route is the Complementary Programme for Physicians with a Medical Degree from Outside the EU/EEA (CPP). The overall intended learning outcome of the programme is congruent with the Swedish medical programme. The Swedish government finances the CPP, which also aims at integrate its participants into Swedish society via work. The CPP has a duration of 10 months and includes theory and practice. The CPP participants undergo four internships in different medical specialties and medical settings (e.g., surgery, psychiatry, paediatrics, emergency, gynaecology, and orthopaedics at hospitals and general practice in primary healthcare). Every participant has a placement in primary healthcare, but none can be guaranteed an internship/placement in every other speciality due to practical limitations. Another route is via Swedish Board of Health and Welfare, which consists of proficiency test, courses in Swedish laws and regulations, and clinical training for 6 months. As a third alternative, MPs may also take the route via the Swedish medical programme (5.5 years at the time of the study).

### Study participants, recruitment, and data collection

Participants were recruited via the CPP in two ways:

(1) The first author visited the campuses and presented the study to participants at three universities during the CPP annual programme in 2017. That year, 65 persons began the programme, 24 of whom volunteered to participate in the study (Interview Set 1). All respondents were invited for a second interview when completing the programme, and 21 accepted the invitation (Interview Set 2). In addition, one MP who had not been interviewed when beginning the programme volunteered for the study. The respondents were invited for a third interview 2.5 years after completing the programme, resulting in four interviews (Interview Set 3a).
(2) The first author emailed an invitation to participate in the study to CPP participants from previous admission years 2012–2016 (n = 278), and 14 volunteered to participate in the study (Interview Set 3b).

The semi-structured interview guide for the first dataset was developed based on knowledge and experience as researchers in the field and research literature, some of which are described in the introduction. The interview guide was iteratively improved during the first interview. For the following sets of interviews, a preliminary analysis of the gathered data was used to inform the interview guides. In Table 1, an overview of the interview sets and themes in the interview guide is displayed.

**Table 1:** Overview of interview sets and themes in the interview guide.

	Interview Set 1	Interview Set 2	Interview Set 3a
	At the beginning of the CPP (2017)	At the end of the CPP (2018)	2.5 years after completing the CPP (2020) Interview Set 3b 3–7 years after completing the CPP (2019)
**Interview guide themes and content**	* Background questions (e.g., age, country of origin and medical education, reasons for migration, work experiences in previous countries and in Sweden) * Differences and similarities in the work as a physician between countries * Professional development and the work as physicians in Sweden (e.g., knowledge gaps to work as a physician in Sweden) * Experienced difficulties for entering the labour market and for working as physicians in Sweden	Follow-up questions from the first interview (life changes, experiences of the CPP) * Professional development and the work as a physician in Sweden (e.g., knowledge gaps to work as a physician in Sweden and any experienced difficulties) * Future career	3a Follow-up questions from the second interview (life changes, experiences in Swedish healthcare) * Professional development and work as a physician in Sweden * Experiences of work life 3b * Background questions (e.g., age, country of origin and medical education, reasons for migration, work experiences in previous countries and in Sweden) * Differences and similarities of work as a physician between countries * Professional development and work as a physician in Sweden
**Number of participants**	24	20 follow-up interviews and 1 new respondent	4 follow-up interviews and 14 new respondents from admission years 2012–2016
**Interview form**	Face-to-face: 23	Face-to-face: 11	Face-to-face: 5
	Digital meeting room: 1	Digital meeting room: 10	Digital meeting room: 4
			Telephone: 9
**Interview length (minutes)**			
Total	1,361	1,138	998
Average	57	54	55
Minimum	27	36	36
Maximum	93	102	73

CPP: Complementary programme for physicians with a medical degree from outside the EU/EEA.

Interviews for Sets 1, 2 and 3a were conducted by the first author. The 14 interviews in Set 3b were conducted by a male medical student in his penultimate year of the medical programme or by the first author. At the time of the first interviews, the first author was a PhD student, and by the time of Interview Set 3a, she had earned her PhD. She is trained in qualitative research methods and has a background in the humanities and social sciences. None of the interviewers had any previous relationship with the respondents.

Most of the interviews were conducted face-to-face. However, due to the pandemic and geographical distances, interviews were also conducted via a digital meeting room and telephone (see Table 1). The interviews were audio-recorded and transcribed verbatim. The interviews ranged from 27 to 103 minutes in length (a total of 3,497 minutes or 58 hours). The interviews were conducted in Swedish.

### Analysis

The data were analysed using qualitative thematic content analysis [30], and we adopted theory of context dependence of knowledge and the different forms of knowledge as a lens [2, 3, 29]. The interview transcripts were initially read for familiarisation by the first author. During re-reading of the transcripts, interviewee demographics, a summary of the interview, meaning units, and preliminary codes were then noted on a blank piece of paper, one for each interviewee, with respective interviewees’ interviews (when applicable) separated on the paper to make comparisons and see any changes over time. Microsoft Excel spreadsheets were used to sort the data, and each interviewee was assigned separate rows for each preliminary code. The first cell in each row denotes the interviewee’s code number. The number of each interviewee’s interviews (no. 1, and when applicable no. 2 and no. 3) was used as column headings, and the interview guide themes (Table 1) were used as sub-column headings. Interviewees’ demographics, the interview summary, meaning units, citations, and preliminary codes were added to the corresponding cells. This method helped manage and keep track of large data-sets. The spreadsheets were read and re-read vertically for one interview at a time and horizontally based on column headings and sub-headings. Preliminary notes on the interpretations were made in separate columns. Codes were refined and categorised into themes in an iterative process; as a final step, the themes were named. During the analysis process, all three authors discussed the material and the interpretations to strengthen the trustworthiness of the results. The authors brought different perspectives to the discussions, stemming from their various scientific backgrounds in humanities (ethnology: LSS and MÖ) and social science (medical education: LSS and TS). No one in the author team had made the same journey as the study participants, and none had worked clinically, but some interviews were conducted by a medical student. The authors (two females and one male) have conducted research in the field for several years and have developed an understanding of the medical context. Once the themes were established, illustrative quotes were chosen from the spreadsheet and translated into English by the author team.

### Ethical considerations

The study was approved by the Swedish Ethical Review Authority and was conducted according to the Helsinki Declaration. Before the interviews, the study participants received written and oral information about the study and gave their written consent to participate. Study information, consent, and interviews were in Swedish, as all study participants had formal qualifications in Swedish. Nevertheless, the fact Swedish was not their mother tongue may have influenced the interviews. Formal qualification in Swedish is, however, a requirement for being accepted into the CPP. There may otherwise be challenges with informed consent when conducting research on migrants due to language as well as concerning migrants’ unfamiliarity with the laws governing data protection, as these may differ between countries. However, the information was formulated as clearly as possible and without complicated language. It was also clear that participation in the study was voluntary, which is supported by the fact that approximately two-thirds of the CPP participants declined to participate in the study. As the group of MPs participating in the CPP was small, we only present the study participants as numbers. Quotes were further anonymised, and the interviewees are not described in more detail for privacy and confidentiality reasons. For this reason, we also chose to present the demographic data at the world region level and did not break them down by country, even though we had the information available. Migrants are often considered a vulnerable group, and we did not want to add any possible inconveniences based on our research.

## Results

Overall, we found that the MPs perceive themselves to have the medical knowledge (encoded knowledge) needed for working in Sweden and that this medical knowledge is thus transferable between medical contexts, with some exceptions that will be described. However, they perceived that they needed to learn how to use their encoded knowledge in the Swedish medical context. The knowledge needed was thus foremost encultured, embedded, embodied, or embrained. Our results are themed as follows: *medical knowledge*; *knowledge of the healthcare system and its variations*; *knowledge of administrative routines*; *understanding the role as a physician*: *interaction and hierarchies between the physician and other healthcare staff*; *understanding the interaction and hierarchies between physicians and patients*, and*; knowledge of the Swedish language*.

The interviewees’ demographics are presented in Table 2.

**Table 2:** The interviewed migrant physicians’ demographics.

	Interview set 1	Interview set 2	Interview set 3
Number of interviews	24	21	18
**Gender**			
Male	14	11	8
Female	11	10	10
**Age (during time of the interview)**			
Mean	33	34	36
Median	29	30	34
Minimum–Maximum (range)	26–50 (24)	27–51 (24)	29–58 (29)
**Region of medical training and degree**			
Africa	4	3	0
Asia	3	2	3
Eastern Europe	5	5	5
Middle East	13	11	4
South America	0	0	6
**Year of medical degree** *			
2010 or before	9	9	8
2011 or later	13	10	10
Minimum–maximum (range)	1991–2015 (24)	1991–2015 (21)	1989–2014 (25)
**Years with medical degree when migrating to Sweden**			
Mean	5	6	3
Median	3	4	2
Minimum–maximum (range)	0–22 (22)	0–22 (22)	0–11 (11)
**Year of migration to Sweden** *			
2010 or before	3	3	6
2011 or later	20	16	12
**Number of years in Sweden (since last migration) during time of the interview**			
Mean	3	5	9
Median	3	4	7
Minimum–maximum (range)	1–8 (7)	2–9 (7)	4–26 (22)
**Position**			
CPP participant	24	21	
Mandatory medical internship position (18–23 months, not having a Swedish medical licence)			6
Temporary position (having a Swedish medical licence)			3
Specialty training			7
Specialist			0

* Information lacking for some participants.

### Medical knowledge

During the first interviews, a few of the MPs mentioned a need to develop or update their medical knowledge, but the same respondents did not mention this in the second interview. However, in the second interview, other MPs expressed that their medical or theoretical knowledge was refreshed during the CPP. In both interview rounds, MPs mentioned a need to develop knowledge about certain specialties, foremost about psychiatry, as this was an unfamiliar area for them. In the second interview set, MPs also expressed that they had developed knowledge and skills related to psychiatry.

“We do not concentrate on psychiatry in my country, as people avoid going to the doctor for these types of problems since it is shameful in our society. Here in Sweden, however, we concentrate on theory but also on how to deal with the patients and about laws such as compulsory care. These were all new things for me”. (MP 24, Interview Set 2)

Additionally, in the second interview set, MPs had learned about context-specific diseases such as inflammatory diseases (i.e., MS and Crohn’s), viral disease tick-borne encephalitis, and gallstones, which some had never encountered before, and one MP also mentioned having experienced more patients seeking medical care for skin changes (such as nevus/moles) than in country of origin and medical training. They also learned about specific drugs. They also mentioned that, in contrast with what they were used to, surgery may be performed without the use of antibiotics in Sweden. Some also mentioned that they, as physicians in Sweden, did not have to prescribe drugs to every patient and were supposed to avoid prescribing when possible (Interview Set 2). Knowledge and experience described as of less use in Sweden concerned, for example, trauma, salmonella, and typhus.

### Knowledge of the healthcare system and its variations

Prior to the CPP, some of the interviewed MPs had worked as medical assistants, nurses or assistant nurses in Sweden, and had developed overall knowledge about the healthcare system from these perspectives. However, MPs also needed to learn about the healthcare system from a physician’s perspective, and over time, this knowledge evolved. In the second interview, some MPs mentioned discovering differences and collaborations between regions, hospitals, and primary care. For example, MPs mentioned that Swedish regions could have different computer systems for patient records, which meant that some had to spend time on internships learning different medical record systems. They also discovered different ways of working in hospitals and primary care in comparison to previous experiences. One MP mentioned, “Here in Sweden, they usually try to treat patients in primary care, and we don’t do the same in [country]” (MP 21, Interview Set 2). In the third interview set, MPs discussed shortcomings in the system, for example, regarding a lack of collaboration between specialties.

In the second interviews, some described that finance seemed to be the priority in Sweden, and that physicians needed to learn to choose cost-effective alternatives, referring to cost for the healthcare system, not for the individual patient. Related to this, an MP in Interview Set 3 mentioned that physicians in Sweden may have a lot of resources and be able to take a lot of samples but must be able to justify their choice of samples. However, another MP from the same Interview Set 3 also mentioned that as a physician in Sweden, medical assessments could be done without having to think about the patients’ financial options.

One expressed need among MPs that was foremost in the first interview set but also during the second was learning about existing resources: for example, available samples and technical equipment such as systems for electronic medical records and X-rays.

During the first interviews, MPs were aware that Sweden has its own laws, regulations, and guidelines, and they mentioned their need to develop knowledge about these. In the second and third interview sets, however, many also described how laws, regulations and guidelines were strictly followed in Sweden, which was characteristic of ‘Swedish physicians’. However, one MP also found that there could be flexibility and small detours from the guidelines for the benefit of the patient. MPs in the second interview set developed detailed knowledge about laws related to access to patient records. One MP who read a patient record during an internship without knowing about the restrictions was informed by a colleague that doing so had been a violation of the law.

### Knowledge of administrative routines

The first round of interviews expressed a need to learn routines. MPs wanted to learn the process for patient management, including enrolment and discharge, how the referral system worked, how to get in touch with specialists and how to write attestations, for example, to the Swedish Social Insurance Agency. Mentioned in the second interview set was how to develop the appropriate way of thinking when managing patients and how to prioritise and structure, for example, for anamnesis and examinations.

Some of what came up during the interviews related to the pace and distribution of time when working as physicians in Sweden. In the first interviews, MPs described their work in Sweden as calm, with few patient encounters during a day, in comparison to their work in previous countries. However, during the second interviews, MPs had developed further insight into the distribution of time between administrational tasks and patient encounters, describing the large amount of time spent on administration in comparison with their experiences in previous countries: ‘You want to have patients and do something more than just sit with the paper, with the patient record’ (MP 24). Working as physicians in Sweden was later described as somewhat stressful. In the third interview set, an MP brought up all the medical certificates that had to be written, which was still perceived as a difficult and time-consuming task:

“I sit and call around to the insurance fund and the employment agency to find out what is required for such medical attestations. And sometimes, it gets wrong. It is a lot of work” (MP, Interview Set 3)

During a third interview, one MP mentioned that she needed to be better at not stressing out or being burned out. Another MP mentioned difficulties handling the high workload. Otherwise, MPs stated that, in general, they managed stress to a great extent and that they themselves worked efficiently.

Issues related to documenting in medical records, including dictating, were expressed across all the interview sets and in most of the individual interviews. Some MPs were not accustomed to using the same medical records as their colleagues or to using digitalised/electronic systems, and to manage the digitalised system for patient records was challenging to learn.

One MP expressed that dictating an epicrisis, or summing up a medical case history was difficult. Therefore, he practised by reading others’ epicrises and highlighted expressions. He described writing and re-reading his own epicrises as time-consuming, compared to the epicrises of physicians educated in Sweden. He explained that they wrote theirs in five minutes. The difficulties of patient records and dictation were also mentioned during the second interview set but were less common than in the first set. The current MP, for example, mentioned that his dictating the epicrisis had improved during the year and that he had decreased the time spent from half an hour to 10 minutes but expressed that he still had a lot to learn. Another MP who was still uncomfortable when dictating epicrises had, since the first interview, found that the length of the epicrises differed depending on the physician, and that physicians had their individual arguments and reasons for this. The MP mentioned that she would keep her epicrises at medium length, as she had learned that it was important to ‘dictate the whole process, thus choosing important things with reasonable and adequate information’ (MP 1, Interview Set 2). She further mentioned that learning this would come from experience. In the second interview set, MPs demonstrated the knowledge that everything had to be documented in the patient records, and therefore wanted to make it good. One MP, however, mentioned that during her internship, she learned from others that she did not need to read the entire patient record; instead, she was told to focus on notes that were most relevant, and those were usually from other physicians. Documenting in medical records, including dictating, remained one of the few difficulties mentioned in the third interview set.

### Understanding the role of the physician, interaction and hierarchies between the physician and other healthcare staff

In general, the interviewed MPs described physicians working in Sweden as accurate, calm, careful, humble, and good at soft skills such as communication. Some MPs mentioned that they needed to manage their temper, for example, by becoming calmer and more patient. Some MPs still mentioned aspects related to their temper in Interview Set 3.

The MPs discovered that they actually did not need to know everything by heart in Sweden and that doing so did not characterise a good physician in the country, unlike the case in their previous countries. Instead, they could seek information elsewhere and when in need, for example, on the internet and in front of patients, if needed. The storage of medical knowledge could thus, to a great extent, be outside the physician but could be consulted ad hoc if necessary. Access to knowledge via the internet, as well as via colleagues, was described as good. Not having to know everything by heart made some interviewees in Interview Set 2 perceive the work in Sweden as easier and less demanding than they were used to, at least regarding that aspect. However, not needing to know everything by heart and instead relying on colleague’s knowledge presupposed asking questions. In time, MPs found that asking questions was important when working as physicians in Sweden, and some mentioned that physicians in Sweden often asked colleagues.

“You should always ask; it has to do with the healthcare culture. In my home country, you should not ask, and asking is a bit shameful. There, when you ask, you are seen as ignorant, but here, it is expected that you need to ask about everything, so you get it right”. (MP 14, Interview Set 2)

Asking questions was for some a threshold to pass, and something they struggled to learn. Some indicated that they were not used to asking questions, as in previous countries, they needed to know everything by heart and not expose any knowledge gaps. In Interview Set 3, one MP described how important it was to ‘discuss with the team and to not be too stubborn, and [how] a physician should be able to argue for one’s cause and motivate’ (MP 10). Physicians in Sweden were described as discussing all the time and with everyone and asking many questions.

In the first interviews, MPs expressed a need to develop knowledge and learn about teamwork, the different professional roles and the distribution of working tasks between the different professions in Sweden—that is, who was doing what. Some discovered that junior physicians did not have any mandate to make any decisions, and that senior physicians decided everything. In general, MPs stated that they were positive about working on teams and with nurses. In the first and second interviews, most MPs mentioned that nurses in Sweden had great competence, which was a relief for them. In the first interviews, they had heard about nurses’ competence, but they later experienced nurses’ competence in action, witnessing how they were part of medical rounds and how physicians took advice from nurses and put their trust in them. However, in the third interview set, MPs also mentioned that they had to accept and learn to respect nurses’ competence.

Social routines in the workplace were learned over time, such as the importance of having lunch and coffee breaks with others in the workplace. MPs in the third interview set described coffee breaks as important for adaptation to the workplace, socialising and team building, as they got to know each other during these events. MPs described it important for peers in Sweden to get to know each other, but many also mentioned that they usually did not seem to socialise after working hours. Further, MPs mentioned that coffee breaks often took place across professional boundaries. An MP in the third interview mentioned being uncomfortable having coffee breaks with nurses and assistant nurses at the beginning of their working career, since it included small talks about their personal lives, which she was not used to from her previous country, where the hierarchy between professions was more noticeable. MPs had to learn what and what not to discuss during coffee breaks. For example, one mentioned that discussing salaries did not seem appropriate.

Teamwork and social routines are related to hierarchies among healthcare staff. Hierarchy was one difference that MPs frequently mentioned during the first interviews, and this relates to both the described teamwork and social routines. They mentioned that relationships between physicians in different positions and between physicians and nurses were less hierarchical in Sweden compared to what they were used to. However, how ‘lesser hierarchy’ was expressed in practice was learned over time; for example, concerning the relationship between physicians asking each other questions and taking advice across their different positions, as one MP expressed in Interview Set 2:

I see that a junior physician can talk to, ask, and give suggestions to a senior physician, and convince the senior physician to change their decision about something. (MP 24, Interview Set 2)

Regarding the relationship between physicians and nurses, one MP in Interview Set 3 expressed having to adapt to the fact that nurses did not necessarily have respect for junior physicians and could question physicians.

### Understanding the interaction and hierarchies between physicians and patients

Mentioned during the first interviews was the need to learn about patient-centred care and to include the patient in treatment decisions, which is the common working method applied in Sweden. Some MPs had very little or no experience with this method, and some had no experience communicating with patients at all, since they had worked in specialties where this was not applicable (e.g., as a surgeon or someone mainly working in laboratories). During the second interview, many MPs expressed that physicians in Sweden are expected not to withhold information from the patient and to explain everything. Some MPs described how they were used to sharing certain information with relatives only and excluding patients. MPs thus learned about patient-centred care and the inclusion of patients. However, in the second and third interview sets, MPs also expressed, and thus had discovered, a need for flexibility regarding patient-centred care and how to communicate with patients. MPs in later interviews mentioned how the approach to patients needed to be individually adapted, although still based on the patients and their wishes and customs, as all patients did not wish to be actively involved in the decision making. In the third interview set, MPs also mentioned the need to increase their knowledge of how to communicate with patients, for example, concerning feedback.

The relationship between physicians and patients was described as less hierarchical in Sweden. During the second interview, one MP reflected on the power relationship that differed between Sweden and their previous country:

It is the patient who decides everything. It was a bit surprising that the physician does not try to convince the patient. If the physician wants to do an examination, but the patient does not want to do it, it is fine […] In [the previous country], is it the physician who decides everything, and not the patient. It is the physician who has power [laughs] over the patient, but here I felt that it is more the patient who has more power. (MP24, Interview Set 2)

Another MP had also learned how to help equalise the power imbalance by lowering his chair to physically end up on the same level as the patient. In time, MPs found that certain questions about the patient’s social and personal life could be relevant when taking their amnesia/medical history. For example, it was described that ‘Here you can ask the patient personal questions, such as “Do you live with someone?”‘ (MP 6, Interview Set 2). However, asking questions relating to personal life in a friendly manner just to socialise was advised against by colleagues. MPs thus developed an understanding of their own roles as physicians in relation to patients.

### Knowledge of the Swedish language

Almost all MPs mentioned aspects relating to language in the first interview set. In the second interview set, language was still mentioned as an aspect that needed to be improved, but only by a third of the interviewees. However, the need to always improve language was mentioned in general terms. Thus, language was not mentioned so much as a difficulty anymore. However, not speaking perfect Swedish remained a concern for some MPs, and some were not yet confident in their language skills. Language remained an area of improvement in the course of work for the MPs in Interview Set 3.

MPs wanted to increase their vocabulary, manage grammar, and improve pronunciation. Difficult to understand were, for example, abbreviations, old-fashioned language and expressions, accents, and slang used by others. Several felt insecure and uncomfortable in different situations without mastery of language, which occurred in all interview sets. Several MPs mentioned that it was a prerequisite to learn, speak, and improve Swedish to work as a physician and practise medicine in Sweden.

**Fig 1:**
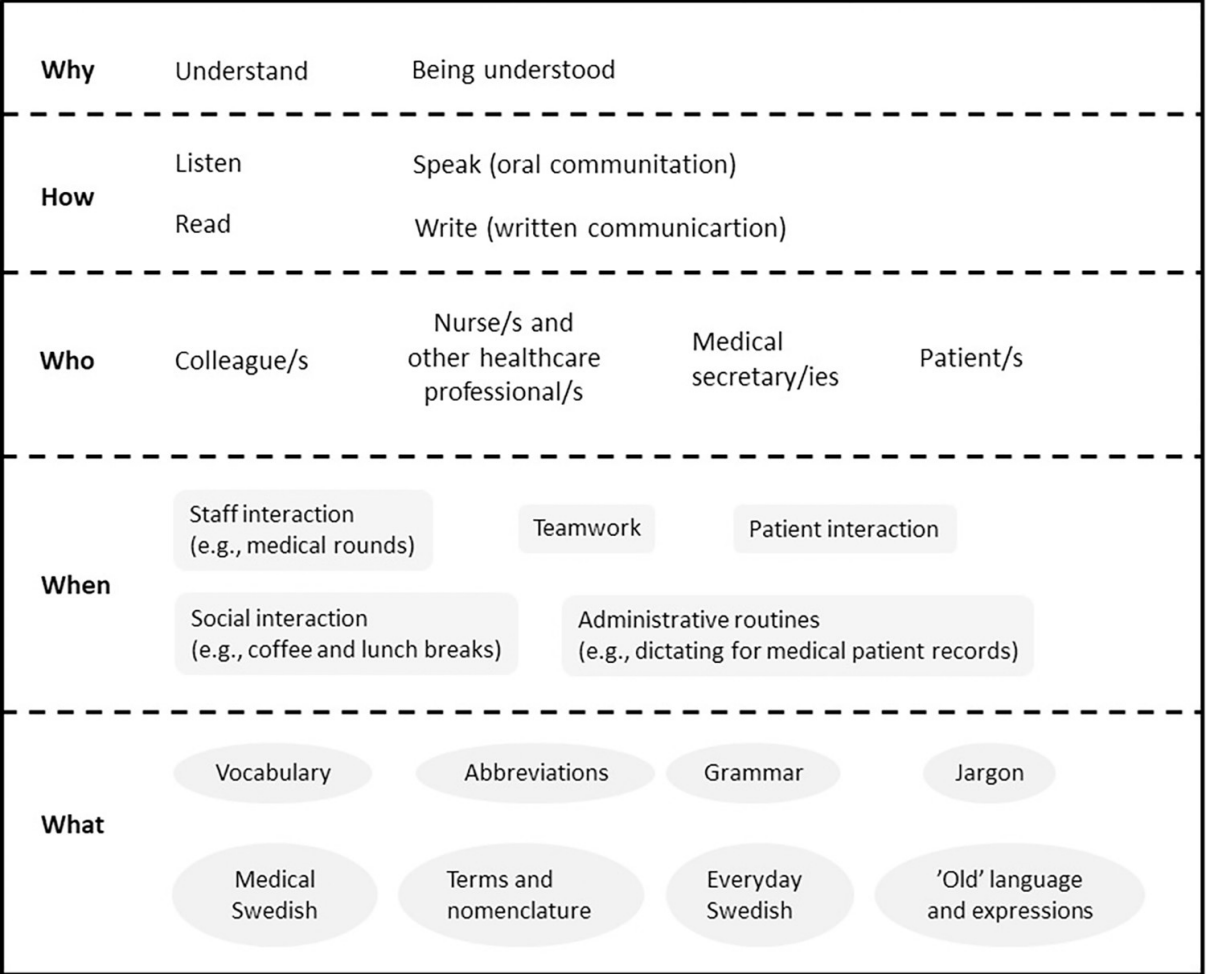
Migrant physicians’ language knowledge needs: Why, how, who, when, and what.

The MPs required knowledge about language to understand, be understood, be able to communicate, carry out working tasks, interact, and manage different situations as a physician. An overview of the interviewee’s knowledge needs and difficulties related to language is presented in Fig 1, and this will be exemplified in the following section.

Language was needed for managing patient encounters and communicating with patients; for example, being able to explain and not having to ask patients questions about the meanings of words. As one MP expressed, ‘Sometimes I feel that it is difficult to explain in a suitable or clear manner to the patient; for example, the patient may not really understand what I mean’ (MP 7, Interview Set 1). Being able to manage language was also about being accepted as a professional by the patients:

When I meet a patient, I have to think about the language now: What should I say to the patient? Will this patient accept that I am a foreign doctor who may sometimes not be able to explain in a good way? (MP 19, Interview Set 1)

Knowledge of language was also needed to be able to participate actively in social routines at work and to have meaningful participation in, for example, medical rounds, a situation in which many spoke quickly at the same time, making it difficult to keep up. Further, language was needed to be able to collaborate and communicate with peers and other healthcare staff, and thus to participate in teamwork and to dictate instructions for others to understand (i.e., peers, other healthcare staff and patients). Dictating and writing patient records were areas perceived as in need of learning and often mentioned as a difficulty during the first interviews which remained an area of concern in the second and the third interview sets. Learning needs and concerns were about nomenclature, finding formulations, using correct words or expressions and being concise.

In the interviews, it was mentioned that different medical specialties and workplaces had their own abbreviations and terminology, thus having different sub-context languages, which sometimes became difficult to understand at first. Not being able to keep up during meetings made some MPs feel stupid, but the feeling decreased over time. Managing language was also about being seen as competent and professional by peers and other healthcare staff. For one MP, it was, for example, important to learn how to write formally and professionally in medical records, since ‘the other physician or nurse will look at what I have written, and maybe they will think that it is not a good way to write and that I then do not know so much’ (MP 8, Interview Set 2). During internships, one MP felt insecure about language and chose to be quiet. She was also used to not speaking unless she had to because of strong hierarchies in her country of origin. Then, she realised that not speaking meant not being able to show and prove knowledge, which also made her insecure and think that others would believe that she knew nothing.

In the third interview set, and except for the need to develop language in general, there was also a need to improve language to be able to give feedback rather than criticism when communicating with peers and other healthcare professionals, such as nurses.

### Longitudinal variations: Key messages

As a short summary of the results, Table 3 presents key messages in each interview round with a focus on longitudinal variation and similarity.

**Table 3:** Key messages in each interview round/longitudinal variations in the results.

Interview set 1	MPs wanted to learn about existing resources, teamwork, patient-centred care, and expressed that Swedish healthcare was less hierarchical than they are used to. Documenting in medical records, including dictating, was frequently mentioned as an area for learning, as well as aspects related to language, which was mentioned by almost everyone as a difficulty.
Interview set 2	MPs had learned about context-specific diseases and developed understanding of interactions such as social routines at work, and how hierarchies are expressed in practice, and reflected upon it. They mentioned that physicians do not need to know everything at heart when working in Sweden, and the importance of asking each other questions. Documenting in medical records, including dictating, was still mentioned as an area in need of learning. Aspects related to language reoccurred but was in this interview set now only mentioned by a third of the interviewees.
Interview set 3	Documenting in medical records, including dictating, reoccurred. Difficulties in writing medical attestants were expressed, and MPs also discussed shortcomings in the healthcare system. The importance of being able to motivate and argue with colleagues about one’s cause. Language reoccurred as an area for learning in this interview set but now more in general terms as something always in need of improvement.

## Discussion

This qualitative quasi-longitudinal study explored the establishment experiences of physicians in the Swedish medical context when trained, and in some cases also having worked, outside the EU/EEA. The aim was to identify the knowledge MPs perceived they needed to develop and did develop over time to work as physicians in Sweden. The knowledge needs and development were themed as follows: medical knowledge; knowledge of the healthcare system and its variations; knowledge of administrative routines; understanding the role as a physician, interaction and hierarchies between the physician; understanding interaction and hierarchies between physicians and patients; and knowledge of the Swedish language. The discussion focuses on and will be based on the theory of the context’s dependence on knowledge and how the different forms of knowledge–encoded, embedded, embrained, embodied, and encultured–are connected [2, 3, 28, 29].

Encoded knowledge is explicit, transferred via text and signs, and is the most mobile of the knowledge forms [3]. We interpret medical knowledge as encoded knowledge, and medicine as a discipline can be considered transnational. The interview material suggested that encoded (medical) knowledge was not too problematic to transfer between contexts, especially with the help of the complementary programme (CPP). However, MPs felt a need to develop knowledge of diseases and conditions common in Sweden, which is context-specific encoded knowledge. However, one could assume that different types of knowledge overlap and are interdependent in an ongoing process in which knowledge is put into use and is under development. If we accept this assumption, it has consequences for MPs’ possibilities to make use of medical knowledge in the new context of Swedish healthcare. To make use of encoded (medical) knowledge and to practise as a physician independently and fully at the destination, we suggest that MPs develop and create knowledge in different areas. In this article, we, for example, mentioned knowledge of the health care system and its variations, vocabularies, routines, and administrative tasks. Most of the knowledge that the interviewed MPs perceived they needed to develop and did develop over time was related to how to perform in their role as physicians in the Swedish context. The possibility of performing as a physician includes two interrelated aspects of being a physician. One is carrying out medical work (being updated and putting medical knowledge into use). The other is finding out how to act and behave in the professional role in a way that is culturally passable in the Swedish context.

We suggest that encultured and embedded knowledge, which might be contextual and place-specific [2, 3, 28], are gateways to being able to, and being allowed to, independently make use of encoded knowledge. The interviewed MPs’ experiences of administrative routines, interaction with colleagues and interaction with patients showed different types of knowledge that were embedded and in need of relearning and/or (cultural) translation. The MPs needed to develop and create knowledge in these areas to turn this knowledge into gateways to the use of embrained medical knowledge.

Thus, our results show how the use of knowledge must be understood as embedded in specific social and cultural contexts, as well as variations in medical systems and organisations. The material also suggests that the possibilities to make use of easily transferable knowledge depend on physicians’ insights into and understandings of context-specific routines and cultural aspects such as hierarchies, relations to nurses, and managing patient-centred care [2, 3, 28].

Knowledge developments are contextual, and processes and outcomes may differ depending on the context rather than the physician, which is probably not unique for MPs. However, MPs have a medical education and sometimes work experience in other national contexts. This makes learning embedded and encultured knowledge especially important as gateways that open up possibilities to use all forms of interdependent knowledge. The knowledge MPs needed to transfer or develop, as well as the gateway knowledge developed, was context-dependent.

The defined areas/gateways, not surprisingly, coincide with what may lead to difficulties, obstacles, and hindrances for MPs. For example, healthcare systems are country-specific, and research has suggested challenges when learning a new healthcare system [6, 15, 16]. Laws, guidelines, and organisations vary regarding when and how to refer patients, the treatments used, and, for example, the names of drugs [6, 15, 16]. Other difficulties that may arise when changing the context, and thus relating to differences between contexts, concern working methods and are related partly to colleagues and partly to patients. The Western way of working is usually based on teams, in which the relationship between physicians and nurses, including hierarchies that exist but are communicated differently than in other contexts and are therefore more difficult to detect, has been described as difficult to adjust to [5, 7–10]. Another Western way of working is the patient-centred approach [7, 8]. Patient-centred care includes elements that may cause difficulties, for example, concerning shared decision making [8] and including patients in decisions during the treatment process [10, 20].

For carrying out medical work, putting medical knowledge to use, and finding out how to act and behave in the professional role in a way that is culturally passable in the Swedish context, language is important. We found that mastering language was important for being able to carry out working tasks, develop relationships, and interact with others in different situations where some type of communication occurred. Managing language was also important for other reasons, such as daring to ask questions, showing competence, feeling competent and being accepted as practising physicians in Sweden. Our results show that the MPs often considered language a difficulty. Language difficulties and barriers for MPs are commonly mentioned in research, and our results are in line with research suggesting that language difficulties may relate to accents, different dialects, medical terminology [8], idioms and nuances [16], abbreviations [20] and engaging in small talk with patients [20]. Language barriers may affect communication between the MP and the patient, other colleagues, and health professional staff [7, 9]. Knowledge of the common language is somewhat fundamental for communication, even though language is just a small part of communication (cf. [26]). Furthermore, knowing and sharing the same language does not imply that individuals can communicate with each other [31] and intercultural communication is even more of a challenge [31]. Medical patient records were not only working tools for the MPs, but they also served as tools for MPs to communicate their knowledge and professionalism, and to do so, they needed to express themselves correctly. In our study, dictating was prominent as a difficulty that remained over time.

We suggest that language was an enabler, and that language may be seen as key to unlocking and passing the gateways for developing knowledge to become an independent practising physician in the new medical context. Language was needed to gain access to encultured and embodied knowledge and to be able to use the encoded (medical) knowledge in practice, thus required to work as a physician in the destination context. The results further show that a destination country’s language is not completely uniform. The development of knowledge in the Swedish language may be based on the other individuals whom the MP encounters during work, as some patients and colleagues, for example, may use old expressions or slang words. Further, specific specialties have varying terminology. Language is thus based in part on the sub-contexts of the destination country context.

### Methodological reflections

Longitudinal studies in this field are unusual and are advocated for. To carry out such a study, building trust and maintaining a relationship with the study participants is important to reduce drop-out [32]. A limitation of this study is that there were only four follow-up interviews in Interview Set 3a; hence, we added an interview set (3b), consequently making this planned longitudinal study quasi-longitudinal one. We can only speculate on why all MPs did not volunteer for a third interview: an ongoing pandemic with great strain on the healthcare and its staff, not feeling successful enough at work or in finding employment, and hence not wanting to talk about such perceived shortcomings, or simply a change in contact details. The interviewees were at a stage of their establishment when a change of location was common and moving to another part in the country or elsewhere, which may also increase drop-out. As the interviewees were distributed all over Sweden, maintaining relationships through, for example, physical locations or spontaneous meetings was not possible. This meant that there was a demographic difference in the last interview set regarding the world regions for medical training, which may have influenced the results. Having worked in different healthcare systems before may have influenced their experiences. The new group of respondents (Set 3b), however, added richness to the data, in that the respondents came from additional countries. A strength of the study is that, although the study participants’ origins and medical degrees were from all over the world, there were still identifiable patterns in the material. Furthermore, the study participants did their internships at different hospitals and in primary care throughout Sweden.

To strengthen the trustworthiness of the results, we used several strategies, such as reflecting upon and describing the research process, the research team members’ roles, and limitations. All results are probably not directly transferable to other contexts, for example, regarding which diseases are context-specific, which medical electronic systems are used, the context-specific language, and specific expressions or jargon. The fact that the study was carried out only in Sweden may be a limitation. However, the overarching themes, and other results (e.g., need to learn routines, understanding interaction and hierarchies, and social routines) and the theoretical discussion are likely to be transferable to other contexts. As described in the discussion, our results are congruent with research conducted in other countries. The strengths of the study include the extensive material and the longitudinal approach. More interviews might, of course, have generated further insights. However, the author team assessed that information power was reached based on the study aim and study participants, having extensive material, an established theoretical framework, and a (quasi) longitudinal approach [33].

Knowledge of medical theory was not a prominent theme in our findings; this may be due to the respondents all participating in the CPP training and hence receiving training to facilitate recertification. MPs who have taken another route to the labour market may thus have other perceptions and experiences.

### Implications for practice and research

The results from this study may be used to develop support to facilitate MPs’ transitions and establishment in a ‘new’ medical context throughout their different establishment phases. Since the study show that knowledge of the language remains an area of learning over time, this might need to be addressed earlier during the establishment phase. This could be done partly through further language courses before a complementary programme and partly within the programme. Further, specific learning activities about dictating could be implemented. Future longitudinal studies could adopt gender or intersectional analysis to identify related patterns, could also focus on physicians trained in specific countries, or include domestically trained physicians for comparisons.

## Conclusion

In our study, we conclude that knowledge of the healthcare system and its variations; knowledge of administrative routines; understanding the role as a physician, interaction and hierarchies between the physician and other healthcare staff; understanding the interaction and hierarchies between physicians and patients; and knowledge of the Swedish language function as gateways that need to be unlocked for practising medical knowledge in the new medical context.

Embedded, embrained, embodied, and encultured knowledge interact and are interdependent, and the different forms of knowledge work as gateways to other forms of knowledge and thus open to each other. However, to pass the gateways and move from a peripheral position to a more central one in the new medical context, managing the common language is important. An enabler for unlocking the gateways is thus language, and we conclude that language is a key to unlocking gateways to practise.
